# Leaf ecological stoichiometry and anatomical structural adaptation mechanisms of *Quercus* sect. *Heterobalanus* in southeastern Qinghai–Tibet Plateau

**DOI:** 10.1186/s12870-024-05010-x

**Published:** 2024-04-24

**Authors:** Aiting Zhou, Bairuixue Ge, Shi Chen, Dingxu Kang, Jianrong Wu, Yanling Zheng, Huancheng Ma

**Affiliations:** 1 Key Laboratory of National Forestry and Grassland Administration on Biodiversity Conservation in Southwest China, Southwest Forestry University, Kunming, 650224 PR China; 2 Key Laboratory of Forest Disaster Warning and Control in Universities of Yunnan Province, College of Forestry, Southwest Forestry University, Kunming, 650224 PR China

**Keywords:** *Quercus* sect. *Heterobalanus*, Ecological stoichiometry, Leaf anatomical traits, Adaptation mechanisms, Pliocene uplift, Hengduan Mountains, Qinghai–Tibet Plateau

## Abstract

**Background:**

With the dramatic uplift of the Qinghai–Tibet Plateau (QTP) and the increase in altitude in the Pliocene, the environment became dry and cold, thermophilous plants that originally inhabited ancient subtropical forest essentially disappeared. However, *Quercus* sect. *Heterobalanus* (*QSH*) have gradually become dominant or constructive species distributed on harsh sites in the Hengduan Mountains range in southeastern QTP, Southwest China. Ecological stoichiometry reveals the survival strategies plants adopt to adapt to changing environment by quantifying the proportions and relationships of elements in plants. Simultaneously, as the most sensitive organs of plants to their environment, the structure of leaves reflects of the long-term adaptability of plants to their surrounding environments. Therefore, ecological adaptation mechanisms related to ecological stoichiometry and leaf anatomical structure of *QSH* were explored. In this study, stoichiometric characteristics were determined by measuring leaf carbon (C), nitrogen (N), and phosphorus (P) contents, and morphological adaptations were determined by examining leaf anatomical traits with microscopy.

**Results:**

Different *QSH* life forms and species had different nutrient allocation strategies. Leaves of *QSH* plants had higher C and P and lower N contents and higher N and lower P utilization efficiencies. According to an N: P ratio threshold, the growth of *QSH* species was limited by N, except that of *Q. aquifolioides* and *Q. longispica*, which was limited by both N and P. Although stoichiometric homeostasis of C, N, and P and C: N, C: P, and N: P ratios differed slightly across life forms and species, the overall degree of homeostasis was strong, with strictly homeostatic, homeostatic, and weakly homeostatic regulation. In addition, *QSH* leaves had compound epidermis, thick cuticle, developed palisade tissue and spongy tissue. However, leaves were relatively thin overall, possibly due to leaf leathering and lignification, which is strategy to resist stress from UV radiation, drought, and frost. Furthermore, contents of C, N, and P and stoichiometric ratios were significantly correlated with leaf anatomical traits.

**Conclusions:**

*QSH* adapt to the plateau environment by adjusting the content and utilization efficiencies of C, N, and P elements. Strong stoichiometric homeostasis of *QSH* was likely a strategy to mitigate nutrient limitation. The unique leaf structure of the compound epidermis, thick cuticle, well-developed palisade tissue and spongy tissue is another adaptive mechanism for *QSH* to survive in the plateau environment. The anatomical adaptations and nutrient utilization strategies of *QSH* may have coevolved during long-term succession over millions of years.

**Supplementary Information:**

The online version contains supplementary material available at 10.1186/s12870-024-05010-x.

## Background

The uplift of the Qinghai–Tibetan Plateau (QTP) was an important geological event of the Cenozoic [1–3]. After two uplifts and two deplanations in the Eocene and late Oligocene up to early Miocene [4–6], most of the plateau remained at low elevation and had a warm and humid tropical/subtropical climate [7, 8]. Vegetation was evergreen-deciduous broad-leaved forests, with Magnoliaceae, Theaceae, Lauraceae, and Fagaceae as the main components of the paleoforest [9]. In the late Pliocene up to early Quaternary, the southeastern QTP was dramatically uplifted nearly 3,500 m, resulting in global climate reshaping, with cooling and drying of the environment and wide changes in vegetation [10–12]. Certainly, the inhospitable conditions of the plateau environment are unfavorable for the development and survival of plants, and thus, the majority species that originally inhabited the ancient subtropical evergreen-deciduous broad-leaved forests essentially disappeared [12]. However, the *Quercus* sect. *Heterobalanus* (*QSH*) of the genus *Quercus* in the family Fagaceae have shown remarkable adaptability to the challenging plateau environment, with nine species of *QSH*: *Q. spinosa*, *Q. pannosa*, *Q. semecarpifolia*, *Q. aquifolioides*, *Q. longispica*, *Q. fimbriata*, *Q. senescens*, *Q. monimotricha*, and *Q. rehderiana* [13]. As altitude increased in the Pliocene, some of the *QSH* species changed from being companion species in mixed forests to dominant or constructive species in many areas of the Hengduan Mountains region [10, 14]. The *QSH* species are ecologically important, because the plants are “umbrella plants” to other plants, animals, and microorganisms on the plateau. However, little is known about the mechanisms that *QSH* species employ adapt to the harsh environment. Ecological stoichiometry can be used to understand how plants adapt to different environments by adjusting the composition and proportion of chemical elements within their bodies [15, 16]. Meanwhile, as the largest exposed vegetative organ of plants, leaves are highly responsive to the environment, and their anatomical structure directly reflects the plant’s adaptive strategies to specific environments [17]. Consequently, the field research has focus on elucidating the adaptive mechanisms of *QSH* survival in plateau environments by studying of ecological stoichiometry and leaf anatomical traits.

Ecological stoichiometry in terrestrial ecosystems is used to examine relations between carbon (C), nitrogen (N), and phosphorus (P) in plants [18, 19]. Analyzing patterns of elemental interactions and constraints within plants, elemental balances, and ecological interactions can help to understand allocation and cycling of plant nutrients and to assess the degree of nutrient scarcity or limitation [20–22]. Carbon is a structural element that is also a substrate and energy source for plant physiological and biochemical processes and growth [23, 24]. Nitrogen and P are the main nutrients for plant growth and in most cases, are also factors limiting plant adaptation and survival [20, 25]. Stoichiometric homeostasis is the theoretical foundation of ecological stoichiometry [26], and the H-value is used to represent the ability to maintain homeostasis in individual plants [27, 28], with higher H-values indicating higher homeostasis and more stable nutrient contents in plants [29]. Strong stoichiometric homeostasis and conservative nutrient use may be essential for species to survive in arid and barren environments [28, 30], and the degree of homeostasis may be highly correlated with species adaptation and ecological strategy [31, 32]. Thus, stoichiometric homeostasis can be used as a predictive tool for plant adaptation to the external environment [18].

Leaves are the plant part most exposed and sensitive to the environment and as a result, have relatively high plasticity and variability. Leaves are the main assimilation organs and physiological activities include photosynthesis, respiration, and transpiration [33–35]. The main anatomical features of leaves are epidermis, mesophyll, and vascular system [36], and differences in those structures can indicate adaptations to specific environments [37, 38]. For example, changes in epidermis thickness can protect leaf tissues against adverse factors and also adjust leaf moisture level and preserve heat [39]. Palisade tissue is the main site of photosynthesis and regulates leaf photosynthetic efficiency by changes in porosity [40]. Spongy tissues with large intercellular spaces can increase gas change. Differences in palisade and sponge tissues can indirectly reflect environmental water status, and thus, the ratio of palisade tissue thickness to sponge tissue thickness (P/S) can reflect leaf resource-use strategies in different habitats [39]. Generally, the higher the P/S ratio reflects the higher the photosynthetic utilization efficiency, which may be one important reason why plants can resist drought stress. Leaves tend to develop morphological structures that are adapted to specific environments [34, 41]. For example, in response to high altitude, evergreen broad leaves usually have relatively thick leaves and palisade and spongy tissues and additional palisade mesophyll layers [33]. Because leaf structure is closely associated with various functions and can reflect long-term adaptability to the environment, it is important to study leaf anatomical characteristics to explore how plants adapt to the environment.

With the rise of the QTP to high altitude, plants suffered from nutrient limitations and a low-moisture environment and leaves were exposed to high radiation. Whereas many plants might have disappeared because they did not develop specific adaptation mechanisms, *QSH* plants may have developed comprehensive adaptation strategies to survive and occupy vacated sites. Hence, this article aims to address the following: (1) Do *QSH* have unique ecological stoichiometric characteristics? (2) Are there any particular anatomical features exhibited by *QSH* leaves? (3) Is there a correlation between the ecological stoichiometry and leaf anatomical structure of *QSH*? This paper attempts to understand the *QSH* nutrient-use strategies that developed in response to the cold, dry, and high-radiation environment of the modern QTP through the ecological stoichiometry and anatomical structures of *QSH* leaves.

## Results

### Leaf nutrient contents and C: N:P ratios of *QSH* life forms

**Fig. 1:**
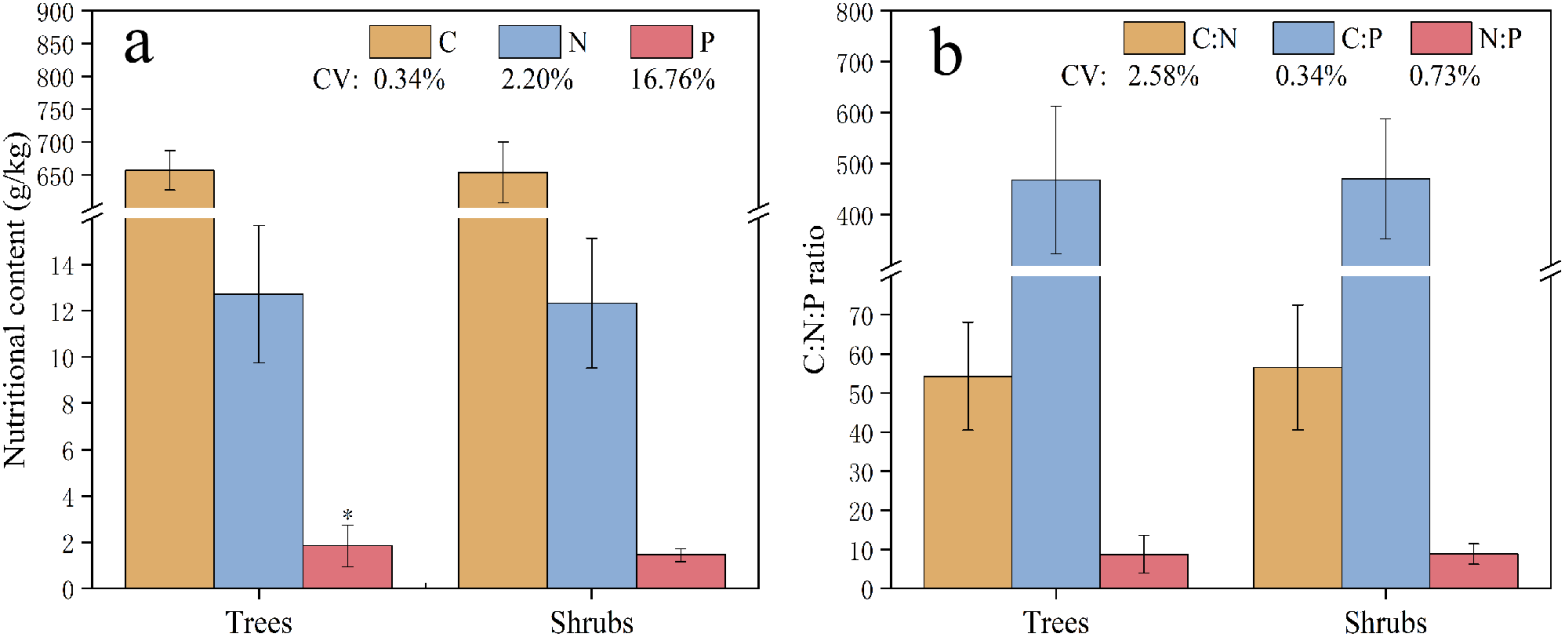
(**a**) Leaf C, N, and P contents and (**b**) stoichiometric ratios in trees and shrubs. The asterisk indicates a significant difference between trees and shrubs (*P* < 0.05). CV is the coefficient of variation. Values are the mean ± SE, *n* ≥ 3

Leaf C, N, and P contents differed slightly among life forms (Fig. 1a and Supplementary Table S1). Mean contents of C and N in leaves of trees were 657.15 g/kg and 12.71 g/kg, respectively, and those in leaves of shrubs were 653.96 g/kg and 12.32 g/kg respectively. Mean P content in leaves of trees (1.84 g/kg) was significantly higher than that in leaves of shrubs (1.45 g/kg), an increase of 26.90% (*P* < 0.05).

In trees leaves, mean nutrient ratios were C: *N* = 54.30, C: *P* = 468.11, and N: *P* = 8.72, whereas in shrubs leaves, mean nutrient ratios were C: *N* = 56.53, C: *P* = 470.34, and N: *P* = 8.81 (Fig. 1b and Supplementary Table S1).

### Leaf C, N, and P contents and stoichiometric ratios of six *QSH* species

**Fig. 2:**
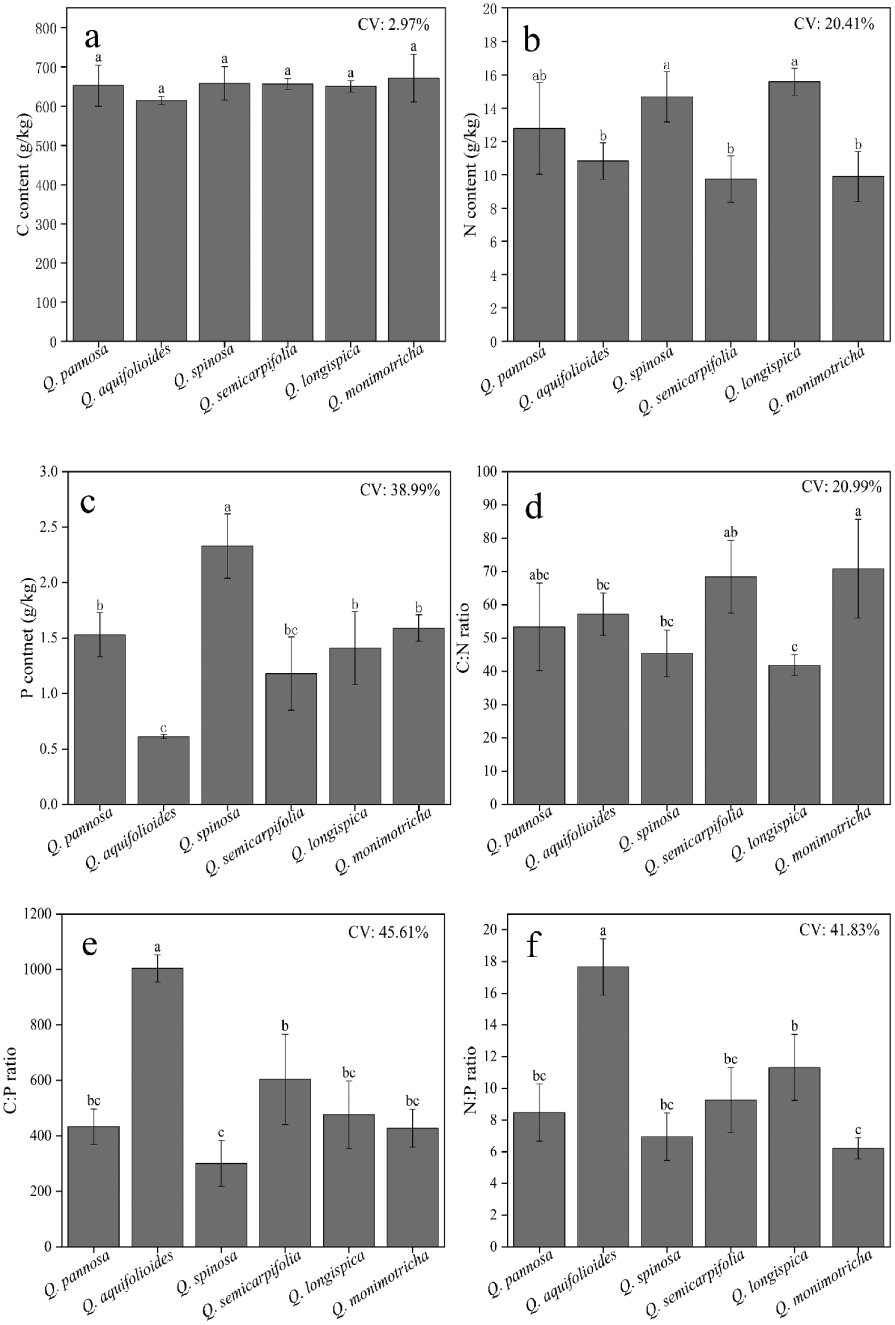
Ecological stoichiometric characteristics in leaves of six *QSH* species. Different lowercase letters indicate significant differences among species (*P* < 0.05). CV is the coefficient of variation. Values are the mean ± SE, *n* ≥ 3

Leaf C, N, and P contents and C: N, C: P, and N: P ratios were significantly different among the six *QSH* species (Fig. 2). Leaf C content in the six species ranged from 614.03 to 671.63 g/kg (Fig. 2a), with a mean of 650.51 g/kg and a coefficient of variation of 2.97%. Leaf C content was in the order *Q. monimotricha* > *Q. spinosa* > *Q. semicarpifolia* > *Q. pannosa* > *Q. longispica* > *Q. aquifolioides*. Leaf N content in the six species ranged from 9.74 to 15.59 g/kg (Fig. 2b), with a mean of 12.25 g/kg and a coefficient of variation of 20.41%. Leaf N content of *Q. aquifolioides*, *Q. monimotricha*, and *Q. semicarpifolia* was significantly lower than that in the other three species (*P* < 0.05). Leaf P content in the six species ranged from 0.61 to 2.33 g/kg (Fig. 2c), with a mean of 1.44 g/kg and a coefficient of variation of 38.99%. *Quercus spinosa* had the highest P content, and there were significant differences among the six species (*P* < 0.05).

Leaf stoichiometric ratios were significantly different among the six *QSH* species (*P* < 0.05; Fig. 2). The mean C: N ratio was 56.18, with ratios varying from 41.84 to 70.84 (Fig. 2d) and a coefficient of variation of 20.99%. The C: N ratios of *Q. monimotricha* was significantly higher (*P* < 0.05) than those of the other species, and *Q. longispica* had the lowest C: N ratio. The mean C: P ratio was 540.80, with ratios varying from 300.55 to 1,003.35 (Fig. 2e) and a coefficient of variation of 45.61%. *Quercus aquifolioides* and *Q. spinosa* had the highest and lowest C: P ratios, respectively. The mean N: P ratio was 9.98, with ratios ranging from 6.21 to 17.66 (Fig. 2f) and a coefficient of variation of 41.83%. The highest N: P ratio was in *Q. aquifolioides*, whereas the N: P ratios of *Q. monimotricha* was significantly lower than those of the other five species (*P* < 0.05).

### Stoichiometric homeostasis regulation coefficient of *QSH* plants

The homeostasis regulation coefficient of C (*1/H*_C_) was less than 0.25 (*P* < 0.1; Table 1), indicating the samples were homeostatic. The value of *1/H*_N_ varied from − 0.1 to 0.28, indicating strictly homeostatic (*P* > 0.10) and homeostatic and weakly homeostatic (*P* < 0.10) regulation. The value of *1/H*_P_ ranged from − 8.97 to 4.80, with higher variation than that of N and indicating strictly homeostatic (*P* > 0.10) regulation. Values of *1/H*_C: N_ and *1/H*_C: P_ consistently indicated homeostatic regulation (*P* < 0.10). The value of *1/H*_N: P_ varied from 0.02 to 1.22. There was greater variability in values of *1/H*_N: P_ than in those of *1/H*_C: N_ and *1/H*_C: P_, and the values indicated strictly homeostatic (*P* > 0.10) and weakly homeostatic (*P* < 0.10) regulation. In conclusion, strong stoichiometric homeostasis characterized the leaves of *QSH* plants, suggesting good adaptability to environmental changes.

**Table 1:** Characteristics of stoichiometric homeostasis in leaves of *Quercus* sect. *Heterobalanus* plants

	Life forms		Species					
	Trees	Shrubs	*Q. pannosa*	*Q. aquifolioides*	*Q. spinosa*	*Q. semicarpifolia*	*Q. longispica*	*Q. monimotricha*
*1/H*_*C*_	−0.01	−0.01	−0.01	−0.02	−0.01	−0.01	−0.01	0
*P*	0	0	0.001	0	0	0	0	0.003
Grade	Homeostatic	Homeostatic	Homeostatic	Homeostatic	Homeostatic	Homeostatic	Homeostatic	Homeostatic
*1/H*_*N*_	0.16	0.15	0.28	0.02	0.26	-0.07	0.27	−0.1
*P*	0.06	0.11	0.067	0.465	0.008	0.023	0.001	0.071
Grade	Homeostatic	Strictly homeostatic	Weakly homeostatic	Strictly homeostatic	Weakly homeostatic	Homeostatic	Weakly homeostatic	Homeostatic
*1/H*_*P*_	4.38	−3.99	−8.97	1.61	4.8	3.75	−5.32	−2.28
*P*	0.007	0.005	0.085	0.005	0.019	0.251	0.486	0.01
Grade	/	/	/	/	/	Strictly homeostatic	Strictly homeostatic	/
*1/H*_*C: N*_	−0.07	-0.06	−0.08	−0.05	−0.14	0	−0.16	0.01
*P*	0.001	0.006	0.005	0.017	0.001	0.014	0.001	0.066
Grade	Homeostatic	Homeostatic	Homeostatic	Homeostatic	Homeostatic	Homeostatic	Homeostatic	Homeostatic
*1/H*_*C: P*_	0.07	0.07	0.06	0.25	−0.04	0.14	0.07	0.05
*P*	0.005	0.005	0.008	0.001	0.004	0.028	0.016	0.022
Grade	Homeostatic	Homeostatic	Homeostatic	Homeostatic	Homeostatic	Homeostatic	Homeostatic	Homeostatic
*1/H*_*N: P*_	0.37	0.32	0.38	1.22	0.12	0.32	0.44	0.02
*P*	0.004	0.007	0.028	0.003	0.156	0.033	0.001	0.108
Grade	Weakly homeostatic	Weakly homeostatic	Weakly homeostatic	/	Strictly homeostatic	Weakly homeostatic	Weakly homeostatic	Strictly homeostatic

*1/H* is the homeostasis coefficient

### Leaf anatomical structural characteristics of six *QSH* species

**Fig. 3:**
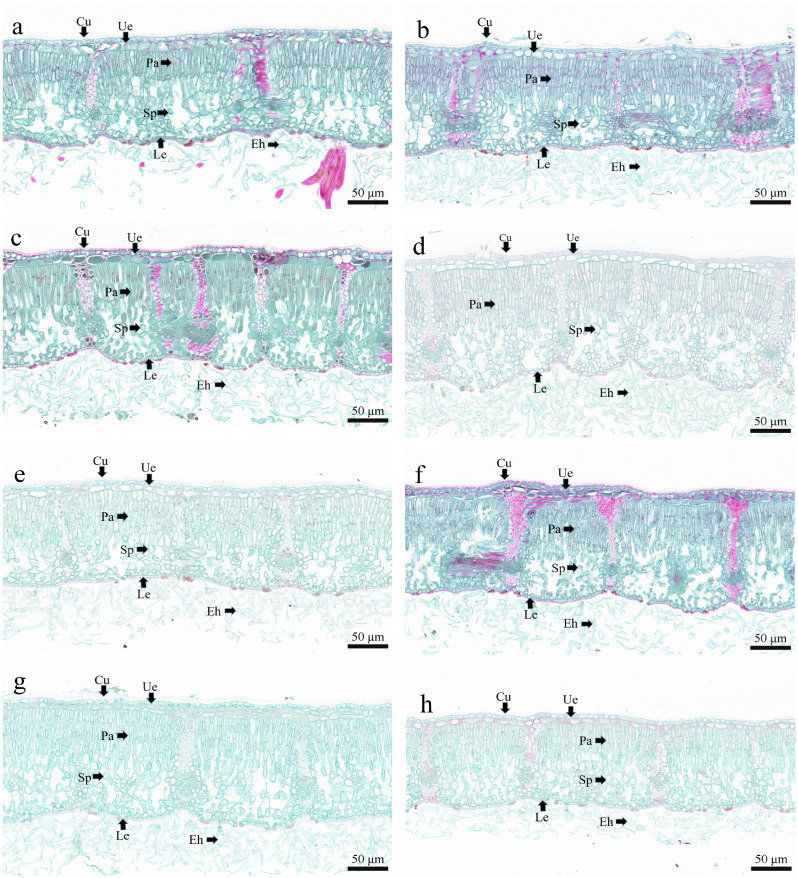
Characteristics of leaf structure of *QSH* plants using optical microscopy (×20). (**a**) Trees, (**b**) shrubs, (**c**) *Q. pannosa*, (**d**) *Q. aquifolioides*, (**e**) *Q. spinosa*, (**f**) *Q. semicarpifolia*, (**g**) *Q. longispica*, and (**h**) *Q. monimotricha*. *Cu* cuticle; *Ue* upper epidermis; *Pa* palisade tissue; *Sp* spongy tissue; *Le* lower epidermis; *Eh* epidermal hair

The *QSH* plants had complex epidermal structure, with tightly arranged epidermal cells, most of which were irregularly shaped, and the leaves were covered with dense epidermal hairs (Fig. 3). Leaf anatomical structure of *QSH* plants differed significantly among life forms and species (*P* < 0.05; Table 2).

**Table 2:** Characteristics of leaf anatomical structures in *Quercus*sect. *Heterobalanus*plants

		CU (µm)	TE (µm)	TP (µm)	TS (µm)	LE (µm)	LT (µm)	P/S ratio	CTR (%)
Life forms	Trees	6.26 ± 0.40*	33.85 ± 3.91*	128.75 ± 5.38*	107.34 ± 6.57*	8.04 ± 1.03*	281.33 ± 9.66*	1.20 ± 0.08	45.84 ± 2.82
	Shrubs	5.64 ± 0.39	29.16 ± 1.62	126.09 ± 3.64	96.29 ± 6.34	7.32 ± 0.42	268.73 ± 6.31	1.31 ± 0.09*	46.95 ± 1.86*
	Mean	5.95	31.50	127.42	101.81	7.68	275.03	1.26	46.40
	CV (%)	7.36	10.54	1.48	7.68	6.65	3.24	6.27	1.69
Species	*Q. pannosa*	5.99 ± 0.52c	29.03 ± 2.30 cd	130.89 ± 6.49c	98.67 ± 8.11c	7.24 ± 0.68bc	279.27 ± 6.88c	1.34 ± 0.13b	46.91 ± 2.80c
	*Q. aquifolioides*	6.81 ± 0.89a	35.14 ± 6.80b	153.28 ± 11.01a	107.41 ± 15.5b	9.01 ± 2.91a	291.85 ± 19.47b	1.46 ± 0.23a	52.74 ± 5.06a
	*Q. spinosa*	6.48 ± 0.74b	40.75 ± 8.47a	119.16 ± 8.87d	98.84 ± 8.83c	9.52 ± 1.54a	319.55 ± 17.65a	1.21 ± 0.14c	44.80 ± 4.15de
	*Q. semicarpifolia*	5.90 ± 0.74c	27.86 ± 2.47d	133.00 ± 7.79c	101.00 ± 9.11c	6.63 ± 1.01c	262.57 ± 15.52d	1.33 ± 0.13b	50.82 ± 4.16b
	*Q. longispica*	6.38 ± 0.68b	30.32 ± 3.82c	138.55 ± 8.97b	114.01 ± 10.67a	7.40 ± 0.75b	266.63 ± 11.57d	1.23 ± 0.14c	43.53 ± 4.18e
	*Q. monimotricha*	4.23 ± 0.51d	27.16 ± 3.03d	107.49 ± 5.44e	89.53 ± 7.93d	6.98 ± 0.78bc	236.79 ± 13.78e	1.21 ± 0.14c	45.53 ± 3.34 cd
	Mean	5.97	31.71	130.39	101.58	7.80	276.11	1.29	47.39
	CV (%)	15.28	16.57	12.12	8.24	15.12	10.20	7.49	7.65

*CU* thickness of upper epidermal cuticle; *TE* thickness of upper epidermis; *TP* thickness of palisade tissue; *TS* thickness of spongy tissue; *LE* thickness of lower epidermis; *LT* blade thickness; *P/S ratio* thickness of palisade tissue to thickness of sponge tissue ratio; *CTR* organizational tightness; *CV* coefficient of variation

Asterisks (*) indicate a significant difference between trees and shrubs according to *t*-test (*P* < 0.05). Different lowercase letters in the same column indicate significant differences among species according to one-way ANOVA (*P* < 0.05). Values are the mean ± SE, *n* ≥ 3

Thickness of upper epidermal cuticle (CU), upper epidermis (TE), palisade tissue (TP), spongy tissue (TS), lower epidermis (LE), and blade thickness (LT) were significantly greater in trees than in shrubs (*P* < 0.05), whereas thickness of palisade tissue to thickness of sponge tissue ratio (P/S ratio) and organizational tightness (CTR) were significantly greater in shrubs than in trees (*P* < 0.05). The coefficient of variation of the different anatomical structures was small and ranged from 1.48 to 10.54%.

Among *QSH* species, CU ranged from 4.23 to 6.81 μm, with a mean of 5.97 μm and a coefficient of variation of 15.28%. The TE ranged from 27.16 to 40.75 μm, with a mean of 31.71 μm and a coefficient of variation of 16.57%. The TP ranged from 107.49 to 153.28 μm, with a mean of 130.39 μm and a coefficient of variation of 12.12%. The TS ranged from 89.53 to 114.01 μm, with a mean of 101.58 μm and a coefficient of variation of 8.24%. The LE ranged from 6.63 to 9.52 μm, with a mean of 7.80 μm and a coefficient of variation of 15.12%. The LT ranged from 236.79 to 319.55 μm, with a mean of 276.11 μm and a coefficient of variation of 10.20%. The P/S ratio ranged from 1.21 to 1.46, with a mean of 1.29 and a coefficient of variation of 7.49%. The CTR ranged from 43.53 to 52.74%, with a mean of 47.39% and a coefficient of variation of 7.65%.

### Correlations between leaf C, N, and P contents and stoichiometric ratios and anatomical traits of *QSH* plants

**Fig. 4:**
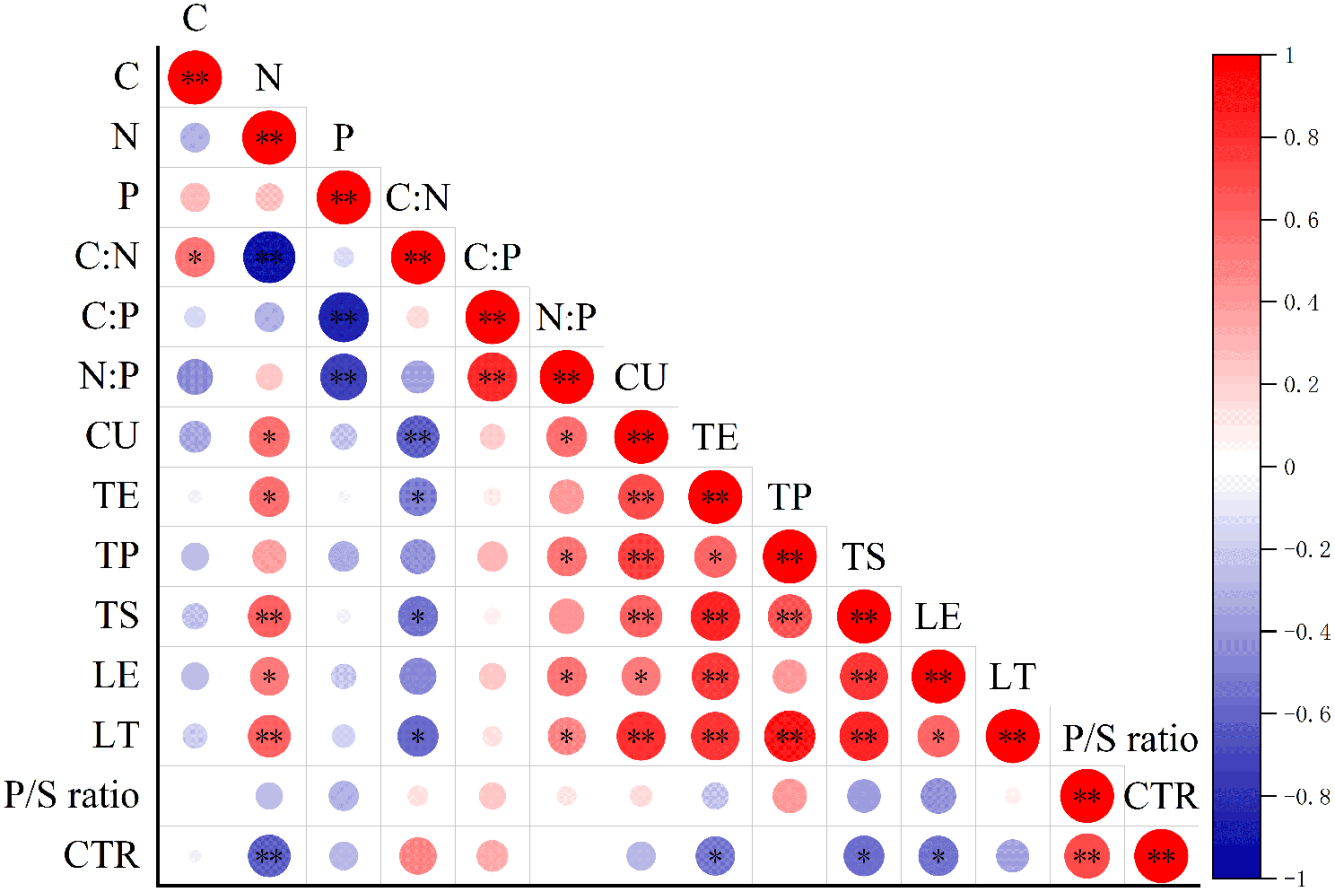
Pearson correlations between leaf C, N, and P contents and stoichiometric ratios and leaf anatomical structures in *QSH* plants. *P < 0.05; **P < 0.01. *CU* thickness of upper epidermal cuticle; *TE* thickness of upper epidermis; *TP* thickness of palisade tissue; *TS* thickness of spongy tissue; *LE* thickness of lower epidermis; *LT* blade thickness; *P/S ratio* thickness of palisade tissue to thickness of sponge tissue ratio; *CTR* organizational tightness

Pearson correlations between C, N, and P contents and C: N, C: P, and N: P ratios and leaf anatomical structures were significant (*P* < 0.05, *P* < 0.01; Fig. 4). Nitrogen was significantly positively correlated with CU, TE, TS, LE, and LT, and the N: P ratio was significantly positively correlated with CU, TP, LE, and LT. Nitrogen was significantly negatively correlated with CTR, and the C: N ratio was significantly negatively correlated with CU, TE, TS, and LT. Notably, the correlations between N, LT, and TS were highly significantly positive (*P* < 0.01), with correlation coefficients of 0.631 and 0.627, respectively.

## Discussion

Research on plant adaptations to the environment has received increasing attention because they are crucial to ecosystem conservation [42]. In terrestrial ecosystems, ecological stoichiometry focuses on organismal energy flow and the balance of multiple chemical elements, which is essential information for exploring plant responses to environmental change, ecological strategies, and use of nutrients that limit plant growth [20, 23, 28, 30]. In addition, because leaf structure is the basis for leaf function, leaves can develop appropriate morphological structures to optimize functions with long-term exposure to the environment. Thus, leaf anatomical traits are also key factors in understanding plant adaptation to the environment [34, 41, 43].

### Leaf nutrient contents of *QSH* plants

Ecological stoichiometry of C, N, and P is an important indicator of C, N, and P status of ecosystems that can be used to understand nutrient characteristics of organisms and to provide important information about N and P limitation [20, 44].

In this study, leaf C contents of trees and shrubs were 41.63% and 40.94% higher, respectively, than the mean global terrestrial leaf C content [45] and 34.86% and 34.20% higher, respectively, than that for *Q. variabilis* in China [46]. Leaf N contents of trees and shrubs were 36.77% and 38.71% lower, respectively, than the global mean value for terrestrial plants [45] and 37.08% and 39.01% lower, respectively, than that for Chinese terrestrial plants [47], and 33.11% and 35.16 lower, respectively, than that for *Q. variabilis* in China [46]. There was a significant difference (*P* < 0.05) in leaf P content between life forms, with P content in tree leaves 26.90% higher than that in shrub leaves (Fig. 1a). The leaf P content of both trees and shrubs was 52.07% and 19.83% higher, respectively, than the mean for Chinese terrestrial plants [47], and 78.64% and 40.78 higher, respectively, than that for *Q. variabilis* in China [46]. The results indicated that the growth rate of trees was faster compared to shrubs, and thus, trees required more P to support their rapid growth, resulting in a higher P content in tree leaves [45, 48, 49].

Leaf C content in the six *QSH* species ranged from 614.03 to 671.63 g/kg, which was 32.33–44.75% higher than the mean global terrestrial leaf C content [45], and 26.01–37.83% higher than the mean for *Q. variabilis* in China [46], and the highest C content was in *Q. monimotricha* (Fig. 2a). Leaf N content ranged from 9.74 to 15.59 g/kg (Fig. 2b), which was 22.44–51.54% lower than the global mean for terrestrial plants [45] and 22.82–51.78% lower than the Chinese mean for terrestrial plants [47], and 17.95–48.74% lower than the mean for *Q. variabilis* in China [46]. The lowest leaf N content was in *Q. semicarpifolia* and *Q. monimotricha*. Leaf P content ranged from 0.61 to 2.33 g/kg (Fig. 2c), with P contents 92.56% higher in *Q. spinosa*, 31.40% higher in *Q. monimotricha*, 26.45% higher in *Q. pannosa*, and 16.53% higher in *Q. longispica* than the mean P content of Chinese terrestrial plants [45]. However, leaf P content of *Q. semicarpifolia* and *Q. aquifolioides* was 2.48% and 49.59% lower, respectively, than the mean in Chinese terrestrial plants [47]. Compared to the average P content of *Q. variabilis* in China [46], with P contents 126.21% higher in *Q. spinosa*, 54.37% higher in *Q. monimotricha*, 48.54% higher in *Q. pannosa*, 14.56% higher in *Q. semicarpifolia*, and 36.89% higher in *Q. longispica.* Conversely, the P content of *Q. aquifolioides* was 40.78% lower than the average for *Q. variabilis* in China [46].

Leaf C content of *QSH* plants was significantly higher than the global mean of terrestrial plants [45] and the mean of *Q. variabilis* in China [46], indicating high C storage capacity. Because of the importance of leaves as assimilation organs, leaf C content can increase to develop defenses against cold and drought and increase overall stress resistance to external environmental factors [50]. Leaf N content in *QSH* plants was lower than global and Chinese means terrestrial plants [45, 47], and also lower than the mean for *Q. variabilis* in China [46], which suggested that N was relatively scarce in the study area. Leaf P content was divided into two categories: (1) a few samples with leaf P content lower than that in Chinese terrestrial plants [47] and lower than the average for *Q. variabilis* in China [46], and (2) most samples with leaf P content higher than the means for Chinese terrestrial plants [47] and *Q. variabilis* in China [46]. The results indicated there was selective absorption of P and thus differences in the nutrient allocation strategies of *QSH* plants in adapting to different habitats. The results in this study are consistent with those of Lin et al. [51], who found that *Larix gmelinii* adopts different nutrient allocation strategies to increase adaptability to different environments. Results also indicated that low temperature leads to an imbalance in the metabolic sink of plants, slowing down plant metabolism [52]. In response, *QSH* plants regulate the metabolic balance by storing P to increase the ability to resist stress. Furthermore, the coefficient of variation of leaf N content was smaller than that of leaf P content (Figs. 1 and 2), which is a result consistent with that of Vitousek [53], who proposed that N content is more stable than P content in leaves. In addition, the results of this study support the proposal of Reich and Oleksyn [54] that N is the major limiting nutrient in relatively young temperate and high-latitude ecosystems.

### Leaf nutrient utilization in *QSH* plants

Leaf C: N and C: P ratios are typically used to indicate nutrient use efficiency, which reflects the ability of plants to use N and P to assimilate C, and the leaf N: P ratio is used to provide key information about nutrient limitations in plants [45, 48, 55]. However, there are different opinions on the N: P ratio threshold. According to Koerselman and Meuleman [56], an N: P ratio < 14 indicates N restriction, an N: P ratio > 16 indicates P limitation, and the N: P ratio between 14 and 16 indicates plant growth is limited by both N and P. In contrast, according to Güsewell [57], a leaf N: P ratio < 10 indicates N limitation, an N: *P* > 20 indicates P limitation, and an N: P ratio between 10 and 20 indicates both N and P limitation. The limiting nutrients in *QSH* plants in this study were identified according to the N: P ratio threshold.

In our study, leaf C: N ratios of trees (54.30) and shrubs (56.53) were 46.36% and 52.37% higher, respectively, than those in plants of a global forest ecosystem [25] and 90.53% and 98.35% higher, respectively, than those in plants of Chinese forest ecosystems [25], and 111.72% and 120.41%, respectively, higher than that of *Q. variabilis* in China [46]. Leaf C: P ratios of trees (468.11) and shrubs (470.34) were 8.75% and 8.32% lower, respectively, than those in plants of Chinese forest ecosystems [19], and 1.06% and 0.58% lower, respectively, than *Q. variabilis* in China [46]. The results suggested that *QSH* trees and shrubs had relatively high N and low P utilization efficiencies, which resulted in an increase in storage of P and a decrease in leaf C: P ratio. Therefore, *QSH* plants might have relatively fast growth rates, especially trees, even in a deteriorated environment [21, 58]. Furthermore, N:P ratios of trees (8.72) and shrubs (8.81) were less than 10, indicating that growth of them was limited by N [56, 57].

Leaf C: N ratios were high among *QSH* species (41.84–70.84), with ratios 12.78–90.94% higher than those in plants of a global forest ecosystem [25] and 46.81–148.56% higher than those in Chinese forest ecosystems [25], and 63.14–176.21% higher than *Q. variabilis* in China [46]. The highest C: N ratio was in *Q. monimotricha* (Fig. 2d). The result indicated that *QSH* plants had high N use efficiency as a strategy to successfully compete in barren environments. Additionally, C:P ratios of *Q. aquifolioides* and *Q. semicarpifolia* were higher than those in plants of Chinese forest ecosystems [19] by 95.58% and 17.72%, respectively, and C: P ratios of *Q. aquifolioides*, *Q. semicarpifolia* and *Q. longispica* were all higher than those of *Q. variabilis* in China [46], which might be the result of relatively high C and low P content in leaves. However, the C: P ratios of the other four species (*Q. monimotricha*, *Q. spinosa*, *Q. pannosa*, and *Q. longispica*) were 7.19–41.41% lower than those in plants of Chinese forest ecosystems [19], and C: P ratios of *Q. monimotricha*, *Q. spinosa* and *Q. pannosa* were all lower than those of *Q. variabilis* in China [46], indicating they had relatively low P utilization efficiency, which were findings consistent with those of life forms. Within Chinese forest ecosystems, *Q. aquifolioides* (N: *P* = 17.66) and *Q. longispica* (N: *P* = 11.32) (Fig. 2f) were co-limited by N and P, whereas the other species were restricted by N [57]. However, according to Koerselman and Meuleman [56], only *Q. aquifolioides* (N: *P* = 17.66) was primarily limited by P, whereas the other species were primarily limited by N. Together with the results of previous studies, in this paper, the framework of Güsewell was considered more accurate [57].

### Leaf stoichiometric homeostasis in *QSH* plants

Stoichiometric homeostasis is a key parameter in ecological stoichiometry [18], and homeostasis regulation reflects potential physiological and biochemical allocation of an organism in response to its surroundings [54, 59]. When external environmental changes lead to nutrient limitations on plant growth, plant can use various physiological mechanisms to improve nutrient availability and use efficiency and maintain stable levels and related functions of nutrients [23]. Plants with strong stoichiometric homeostasis are relatively conservative in nutrient use, whereas those with relatively weak homeostasis can flexibly use nutrients when abundant [28, 60]. Thus, the level of stoichiometric homeostasis can reveal plant ecological adaptation mechanisms [18, 32, 59]. In this study, *1/H* was used as a quantitative index to determine the level of homeostasis, and stoichiometric homeostasis of *QSH* leaves was classified as strictly homeostatic, homeostatic, and weakly homeostatic (Table 1). However, *1/H*_P_ and *1/H*_N: P_ values of some species were not within the range to evaluate homeostasis. The most likely explanation for those results might be that a constant *C* that close to the true value was not identified or that the dependent variable *Y* was less than or equal to the constant *C*, resulting in *P* < 0.10 and *1/H* > 1. Furthermore, in this study, there were differences in absorption, storage, and utilization of N and P among different life forms and species. The differences might be driven by the life history or specific habitats of plants. The homeostasis level of trees was weaker than that of shrubs, and the high homeostasis of *Q. aquifolioides* might be one of the important factors for its wide distribution in the Hengduan Mountains region (author observation). The stoichiometric characteristics and homeostasis of C, N, and P contents and stoichiometric ratios in leaves led to the conclusion that *QSH* plants had strong stoichiometric homeostasis and were conservative in use of nutrients [28, 59]. In addition, the species with the highest stoichiometric homeostasis had the most constant response to environmental changes [27, 28, 59]. The high degree of stoichiometric homeostasis might help explain how *QSH* plants adapted to the arid, cold, and nutrient-poor environment of the plateau.

### Leaf anatomical structures in *QSH* plants

In response to the environment, leaves are the most sensitive and plastic organs during plant growth and development, and because leaf morphological structure is the foundation of leaf functions, leaf structural characteristics are good indicators of plant adaptability to the environment [38, 41, 61, 62]. In the Hengduan Mountains, with perennial low temperatures, drought, and intense light environment, the leaves of *QSH* plants should form structures compatible with that environment. In this study, the differences in anatomical traits among different life forms and species of *QSH* plants reflected different survival strategies in specific growth environments. However, in general, leaves of *QSH* plants had dense epidermal hairs and thick cuticle and upper epidermis (Table 2; Fig. 3), which can effectively reduce water evaporation, mitigate UV radiation, and resist pathogen invasion [33, 34]. Thus, leaf structure was an important manifestation of *QSH* plant adaptation to harsh environments. The compound epidermal structure of *QSH* plants is unique in the *Quercus* genus, and it benefited *QSH* plants and allowed them to become winners in the plateau competition [63]. Moreover, the coefficients of variation of different anatomical structures of *QSH* leaves were relatively small (Table 2). One explanation might be that the ecological adaptability of *QSH* plants to the environment was manifested in a relatively stable morphological structure, which may also be a systematic evolutionary trait. Leaf traits can be interpreted as one reason why only *QSH* plants survived after the severe uplift of the QTP in late Pliocene up to early Quaternary. Additionally, the activity of photosynthesis-related enzymes is restricted under low temperatures, which results in a decrease in photosynthetic rate [52, 61]. Palisade tissue is the main site of photosynthesis, and large cellular spaces in sponge tissue increase gas exchange [34, 36]. In this study, the *QSH* plants had well-developed palisade tissue and sponge tissue, which increased the number of chloroplasts and CO_2_ diffusion rate, compensating for the short photosynthesis period on the plateau [39, 52, 64]. The P/S ratio was greater than one and indicated that *QSH* plants were typical drought-resistant plants. The CTR can be used to evaluate plant cold resistance [52]. Thus, the high CTR and increases in palisade tissue in leaves among different life forms and species of *QSH* plants suggested reductions in damage caused by low temperatures, drought, and UV radiation in the Hengduan Mountains. The P/S ratio and CTR of shrubs were higher than those of trees, and *Q. aquifolioides* had the highest values among the six species (Table 2), which was consistent with the results of stoichiometric homeostasis. This result was considered to be the outcome of coevolution between anatomical traits and nutrient use in developing plant adaptation strategies.

### Correlations between leaf stoichiometric and anatomical traits in *QSH* plants

Correlations between leaf stoichiometric and anatomical structural traits of *QSH* plants at 16 plots in the Hengduan Mountains were analyzed, and N content and C: N and C: P ratios were significantly correlated with anatomical structures (*P* < 0.05, *P* < 0.01; Fig. 4). In the previous section, it was concluded that leaf N content in *QSH* (Figs. 1 and 2) life forms and species was lower than the mean N levels of terrestrial plants globally and in China [45, 47], which indicated that N was scarce in the study area. Leaf N content was highly positively correlated (*P* < 0.01) with blade thickness (*r* = 0.631) and sponge tissue (*r* = 0.627) (Fig. 4). The low N content indicated that *QSH* leaves were relatively thin overall, which might be the result of high leathering and lignification of leaves. The conclusion in this study differs from that in previous studies in which high light intensity led to an increase in blade thickness [65, 66]. Thinning leaves is beneficial for plants, because dry matter of the same quality can be used to increase leaf area, thereby increasing solar energy capture capacity and carbon accumulation [38] and ultimately causing high C: N ratios and strengthened leaf fibers. Consistent with that conclusion, in this study, *Q. monimotricha* leaves were the thinnest (236.79 μm; *P* < 0.05) but had the highest C content and C: N ratio, which indicated leaf leathering and lignification increased to improve plant resistance. Simultaneously, the thin spongy tissue, which corresponded to low leaf N content, suggested that the leaves had well-developed palisade tissue and high organizational tightness, which reduced the damage caused by strong light and UV radiation and helped *QSH* plants adapt to cold, arid, and nutrient-poor habitats. Correlation analysis further indicated the potential coevolution of nutrient use and leaf anatomical traits in adaptation strategies of *QSH* plants to the plateau environment.

## Conclusion

In this study, leaf nutrient contents, stoichiometric ratios, and anatomical traits of *QSH* plants were examined in the Hengduan Mountains, southeastern QTP, Southwest China. Stoichiometric characteristics of different *QSH* life forms and species were different and therefore indicated unique strategies to adapt to the plateau environment. Increases in leaf C content caused high C: N ratios and increases in leaf P content caused low C: P ratios. Growth of *QSH* plants was primarily limited by N, except in *Q. aquifolioides* and *Q. longispica*, which were limited by both N and P. Although stoichiometric homeostasis of trees was weaker than that of shrubs and there were some differences among species, the stoichiometric homeostasis of *QSH* plants was very strong overall, indicating a strategy to address the nutrient limitations to some extent. In addition, leaf anatomical traits were different among different life forms and species of *QSH* plants. Generally, the morphological structure of *QSH* plant leaves was adapted to the plateau environment, with thick cuticle, compound epidermal layer, dense epidermal hairs, and developed palisade tissue reducing water transpiration and damage from UV radiation. More importantly, *QSH* plants increased leathering and lignification by thinning leaves. Furthermore, leaf ecological stoichiometric and anatomical structural traits were significantly correlated, suggesting that *QSH* plants jointly regulated the different functional traits in response to the unfavorable environmental conditions in the Hengduan Mountains.

## Methods

### Study area

The study was conducted in the Hengduan Mountains in southeastern QTP, Southwest China (26°08′54″–30°42′49″N, 99°09′28″–101°29′15″E), which are a world*-*renowned biodiversity hotspot [67]. The Hengduan Mountains are mainly influenced by the confluence of air currents of southeast Pacific monsoon, southwest Indian Ocean monsoon, and south branch rapids of the high-altitude westerly circulation of the Tibetan Plateau, which result in an overall pattern of dry winters and rainy summers. Average elevation is greater than 3,500 to 4,000 m, annual average temperature is 14 ℃ to 16 ℃, over 85% of total precipitation occurs between June and August, and the soil is shallow, barren, and with high gravel ratio and low water retention ability. The vertical spectrum of the climate is obvious, and the vegetation zones from bottom to top of a mountain are successively composed of trees, shrubs, and meadows, with the primary species including *Larix gmelinii*, *Pinus densata*, *Picea asperata*, *Abies fabri*, *Quercus* sp., *Juniperus recurva*, *Rhododendron simsii*, *Spiraea salicifolia*, *Lonicera japonica*, and *Rhodiola rosea*. The *QSH* plants are constructive or dominant species on the sunny slopes of many parts in the Hengduan Mountains region.

### Sampling and measurements

**Fig. 5:**
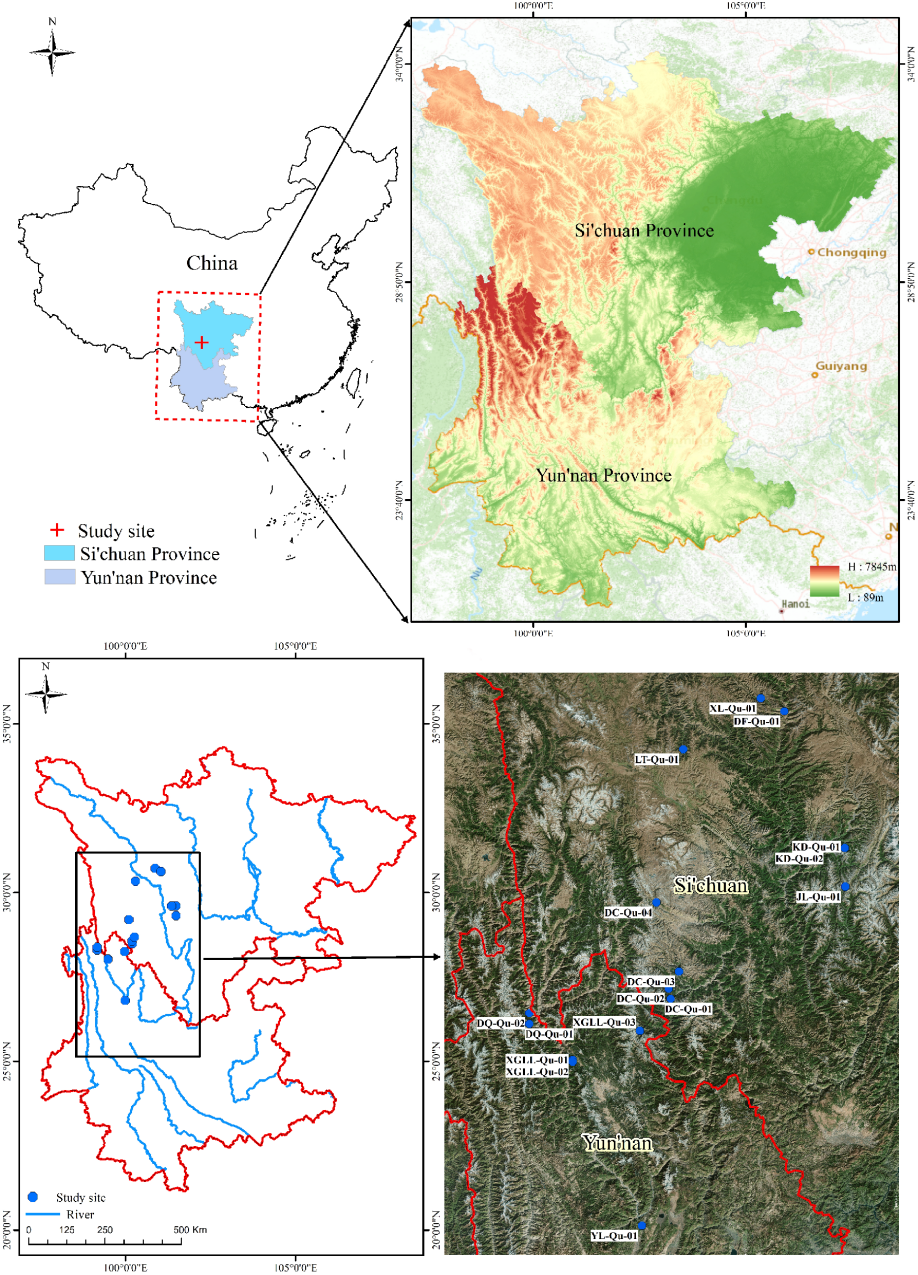
Geographic location and map of the study area

Samples of *QSH* plants were collected from southwest China in August 2022 (Fig. 5). Voucher specimens have been deposited in the herbarium of the Southwest Forestry University. The experiment involved 16 sample plots, including a total of six species of *QSH*: *Q. pannosa*, *Q. aquifolioides*, *Q. spinosa*, *Q. semecarpifolia*, *Q. longispica*, and *Q. monimotricha.* These species include two life forms: trees and shrubs (Table 3). Sample sites were distant from anthropogenic disturbances and had pure forests of *QSH* plants and sunny slopes. Three uniform trees of the same species were randomly selected in each plot, and fully exposed and mature leaves were collected in the outer parts of the same canopy of each tree in east, south, west, and north directions using a branch shear. Soil samples (depth: 1–10 cm) were collected from each sample tree along the directions of east, south, west, and north. The leaves were mixed separately into a composite sample for each plot and then placed in labeled, sealed bags and taken to the laboratory. In the lab, leaf samples were boiled at 105 °C for 30 min and then dried at 65 °C to constant weight and ground into powder and passed through a 100-mesh sieve for chemical analysis. Carbon content was measured by a potassium dichromate external heating method [68], and N and P contents were measured with an automatic Kjeldahl apparatus method (NY/T2419-2013) and a vanadium molybdate blue colorimetric method (NY/T2421-2013), respectively. Soil C contents was measured by an elemental analyzer method [69], and soil N and P contents determined using the Kjeldahl method (HJ717-2014) and the forest soil phosphorus determination method (LY/T1232-2015-3), respectively. Three replicates were set for every measurement.

**Table 3:** Voucher information for *Quercus* sect. *Heterobalanus* plants

Species	Voucher	Locality	Altitude (m)	Life forms
*Q. pannosa*	YL-Qu-01	Lijiang, Yunnan, China	2494	Shrubs
*Q. pannosa*	DQ-Qu-02	Deqin, Yunnan, China	3357	Shrubs
*Q. pannosa*	XGLL-Qu-01	Shangri-La, Yunnan, China	2994	Shrubs
*Q. pannosa*	DF-Qu-01	Daofu, Sichuan, China	3559	Shrubs
*Q. pannosa*	JL-Qu-01	Jiulong, Sichuan, China	4007	Shrubs
*Q. aquifolioides*	DQ-Qu-01	Deqin, Yunnan, China	3357	Trees
*Q. spinosa*	XGLL-Qu-02	Shangri-La, Yunnan, China	3558	Trees
*Q. spinosa*	XGLL-Qu-03	Shangri-La, Yunnan, China	3904	Trees
*Q. spinosa*	XL-Qu-01	Xinlong, Sichuan, China	3474	Shrubs
*Q. semicarpifolia*	DC-Qu-01	Daocheng, Sichuan, China	3309	Trees
*Q. semicarpifolia*	KD-Qu-01	Kangding, Sichuan, China	4085	Shrubs
*Q. longispica*	DC-Qu-02	Daocheng, Sichuan, China	4101	Trees
*Q. longispica*	DC-Qu-04	Daocheng, Sichuan, China	3976	Shrubs
*Q. monimotricha*	DC-Qu-03	Daocheng, Sichuan, China	3631	Shrubs
*Q. monimotricha*	LT-Qu-01	Litang, Sichuan, China	3537	Shrubs
*Q. monimotricha*	KD-Qu-02	Kangding, Sichuan, China	3142	Shrubs

The homeostasis coefficient *1/H* was used to indicate the strength of plant stoichiometric homeostasis [48] and was calculated as follows:

1 $$Y=C{X}^{\frac{1}{H}}$$

2 $$\frac{1}{H}=\frac{lgY-lgC}{lgX}$$

where *Y* is the C, N, or P content or C: N, C: P, or N: P ratio in leaves; *X* is the C, N, or P content or C: N, C: P, or N: P ratio in soil (Supplementary Table S2); and *C* is a constant. Values for *H* and *C* are obtained according to the regression between *Y* and *X*, and *1/H* is the regression slope between log *X* and log *Y*, with an absolute value of 0.00 to 1.00. According to one-tailed tests with α = 0.10, when a regression relation was not significant (*P* > 0.10), the plant was defined as “strictly homeostatic”, whereas when the regression relation was significant (*P* < 0.10), stoichiometric homeostasis was divided into homeostatic (0 < *1/H* < 0.25), weakly homeostatic (0.25 < *1/H* < 0.5), weakly plastic (0.5 < *1/H* < 0.75), and plastic (*1/H* > 0.75) [60].

Collected fresh leaf samples were cut into 1 cm × 1 cm pieces and immediately put into FAA solution (100 mL of FAA = 90 mL of 70% ethanol + 5 mL of 37% formaldehyde + 5 mL of 99.5% glacial acetic acid). Fixed materials were dehydrated in ethanol, made transparent in xylene, embedded in paraffin, and then sliced using a microtome (Leica RM 2016, Shanghai, China) to a section thickness of 8–10 μm. Safranine O-fast green staining [70] was performed after specimens were dried, and then, specimens were sealed using neutral resin and photographed with an optical microscope (Leica DM750, Weztlar, Germany). Data were obtained by CaseViewer 2.4 (Budapest, Hungary). Ten measurements were performed per leaf, and the mean was obtained. The measured parameters were thickness of upper epidermal cuticle (CU), thickness of upper epidermis (TE), thickness of palisade tissue (TP), thickness of spongy tissue (TS), blade thickness (LT), and thickness of lower epidermis (LE). The P/S ratio (P/S = TP/TS) and organizational tightness (CTR = TP/LT × 100) were obtained by calculation.

### Statistical analyses

SPSS software (v20.0, IBM, Armonk, NY, USA) was used to process and analyze data, and figures were made using Origin (v2023b, OriginLab Corporation, Hampton, USA). Significant differences in leaf C, N, and P contents and C: N, C: P, and N: P ratios and in leaf anatomical structures among life forms and species were analyzed by one-way ANOVA and *t*-test, respectively. The significance level was set at 5%. Pearson correlation analysis was performed to analyze the relationship between leaf stoichiometric and anatomical traits. There were three replicates in each measurement. Data are represented as mean ± standard deviation.

### Electronic supplementary material

Below is the link to the electronic supplementary material.

Supplementary Material 1

Supplementary Material 2

## Data Availability

The data sets used and/or analyzed during the current study are available from the corresponding author on reasonable request.

## Abbreviations

QTP: Qinghai–Tibet Plateau
*QSH*: *Quercus* sect. *Heterobalanus*
C: carbon
N: nitrogen
P: phosphorus
C: N carbon to nitrogen ratio
C: P carbon to phosphorus ratio
N: P nitrogen to phosphorus ratio
CV: the coefficient of variation
*1*/*H*: homeostasis coefficient
*1*/*H*_C_: homeostasis regulation coefficient of carbon
*1/H*_N_: homeostasis regulation coefficient of nitrogen
*1/H*_N_: homeostasis regulation coefficient of phosphorus
*1/H*_*C*_: _N_ homeostasis regulation coefficient of carbon to nitrogen ratio
*1/H*_*C*_: _*P*_ homeostasis regulation coefficient of carbon to phosphorus ratio
*1/H*_*N*_: _*P*_ homeostasis regulation coefficient of nitrogen to phosphorus ratio
CU: thickness of upper epidermal cuticle
TE: thickness of upper epidermis
TP: thickness of palisade tissue
TS: thickness of spongy tissue
LE: thickness of lower epidermis
LT: blade thickness
P/S: thickness of palisade tissue to thickness of sponge tissue ratio
CTR: organizational tightness
Cu: cuticle
Ue: upper epidermis
Pa: palisade tissue
Sp: spongy tissue
Le: lower epidermis
Eh: epidermal hair
