# Supplementary material for: Leaf ecological stoichiometry and anatomical structural adaptation mechanisms of Quercus sect. Heterobalanus in southeastern Qinghai–Tibet Plateau
Source: BMC Plant Biol. 2024 Apr 24;24:325. doi: 10.1186/s12870-024-05010-x (PMC11040857; doi:10.1186/s12870-024-05010-x)
Supplement: Supplementary file 1 — Supplementary Material 1 [file 12870_2024_5010_MOESM1_ESM.docx]

**Supplementary Table S1 Characteristics of C, N, P, and C:N:P in leaves of *QSH* plants**

|  |  | C (g/kg) | N (g/kg) | P (g/kg) | C:N | C:P | N:P |
| --- | --- | --- | --- | --- | --- | --- | --- |
| Life forms | Trees | 657.15±29.93 | 12.71±2.96 | 1.84±0.90* | 54.30±13.77 | 468.11±144.69 | 8.72±4.81 |
|  | Shrubs | 653.96±46.36 | 12.32±2.80 | 1.45±0.27 | 56.53±15.94 | 470.34±118.29 | 8.81±2.62 |
|  | Mean | 655.56 | 12.52 | 1.65 | 55.42 | 469.23 | 8.77 |
|  | CV (%) | 0.34 | 2.20 | 16.76 | 2.85 | 0.34 | 0.73 |
| Species | *Q. pannosa* | 652.56±52.29a | 12.78±2.75ab | 1.53±0.20b | 53.38±13.11abc | 433.30±63.98bc | 8.46±1.80bc |
|  | *Q. aquifolioides* | 614.03±10.63a | 10.82±1.09b | 0.61±0.02c | 57.19±6.33abc | 1003.35±49.48a | 17.66±1.78a |
|  | *Q. spinosa* | 657.96±42.88a | 14.68±1.51a | 2.33±0.29a | 45.40±7.06bc | 300.55±82.78c | 6.94±1.5bc |
|  | *Q. semicarpifolia* | 656.45±14.31a | 9.74±1.39b | 1.18±0.33bc | 68.42±10.87ab | 603.88±162.81b | 9.26±2.06bc |
|  | *Q. longispica* | 650.41±14.57a | 15.59±0.81a | 1.41±0.33b | 41.84±3.12c | 476.14±121.83bc | 11.32±2.09b |
|  | *Q. monimotricha* | 671.63±61.11a | 9.89±1.50b | 1.59±0.12b | 70.84±14.83a | 427.56±68.28bc | 6.21±0.67c |
|  | Mean | 650.51 | 12.25 | 1.44 | 56.18 | 540.8 | 9.98 |
|  | CV (%) | 2.97 | 20.41 | 38.99 | 20.99 | 45.61 | 41.83 |

Asterisks (*) indicate a significant difference between trees and shrubs according to *t*-test (*P* < 0.05). Different lowercase letters in the same column indicate significant differences among species according to one-way ANOVA (*P* < 0.05). Values are the mean ± SE, *n* ≥ 3. CV is the coefficient of variation.
