# Supplementary material for: Leaf ecological stoichiometry and anatomical structural adaptation mechanisms of Quercus sect. Heterobalanus in southeastern Qinghai–Tibet Plateau
Source: BMC Plant Biol. 2024 Apr 24;24:325. doi: 10.1186/s12870-024-05010-x (PMC11040857; doi:10.1186/s12870-024-05010-x)
Supplement: Supplementary file 2 — Supplementary Material 2 [file 12870_2024_5010_MOESM2_ESM.docx]

**Supplementary Table S2 Characteristics of C, N, P, and C:N:P in soil of *QSH* plants**

|  |  | C (g/kg) | N (g/kg) | P (g/kg) | C:N | C:P | N:P |
| --- | --- | --- | --- | --- | --- | --- | --- |
| Life forms | Trees | 65.88±20.70 | 3.02±1.18 | 1.17±0.33 | 22.66±5.02 | 60.00±21.73 | 2.64±0.91 |
|  | Shrubs | 48.15±18.83 | 2.65±1.34 | 0.90±0.36 | 19.44±5.56 | 58.43±23.09 | 3.13±1.40 |
| Species | *Q. pannosa* | 39.43±14.75b | 1.92±0.81b | 0.94±0.53a | 21.46±7.27ab | 50.96±28a | 2.39±1.28a |
|  | *Q. aquifolioides* | 54.62±1.13ab | 1.84±0.07b | 0.77±0.02a | 29.74±1.61a | 71.15±3.50a | 2.39±0.06a |
|  | *Q. spinosa* | 66.33±28.19ab | 3.55±1.22ab | 1.22±0.27a | 18.36±1.48b | 57.22±27.32a | 3.06±1.30a |
|  | *Q. semicarpifolia* | 61.98±13.13ab | 3.68±0.25ab | 1.12±0.49a | 17.01±4.71b | 58.23±13.93a | 3.68±1.84a |
|  | *Q. longispica* | 78.39±19.45a | 4.12±2.29a | 0.98±0.10a | 20.97±6.96ab | 79.15±11.70a | 4.09±1.92a |

Different lowercase letters in the same column indicate significant differences among species according to one-way ANOVA (*P* < 0.05). Values are the mean ± SE, n ≥ 3.
